# Supplementary material for: Dual nature of magnetic dopants and competing trends in topological insulators
Source: Nat Commun. 2016 Jun 27;7:12027. doi: 10.1038/ncomms12027 (PMC4931223; doi:10.1038/ncomms12027)
Supplement: Supplementary Information — Supplementary Figures 1-3, Supplementary Note 1 and Supplementary References [file ncomms12027-s1.pdf]

<u>**Supplementary Figures:**</u>

Supplementary Figures 1 and 2 – Theoretical modeling

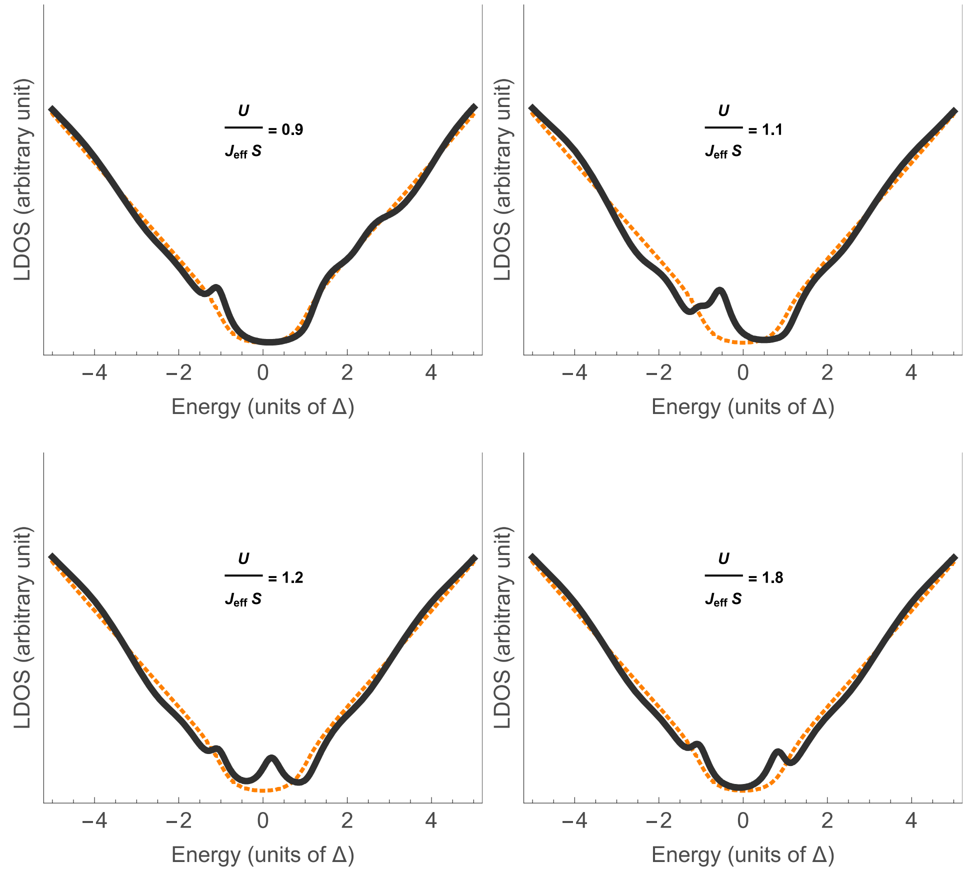

Supplementary Figure 1: The LDOS at a location with many nearby impurities, for various positive ratios of the scalar potential impurity strength, U, and the residual impurity exchange strength, $J_{\text{eff}}\mathbf{S}$. Δ is the mean field mass of the Dirac gas, which arises due to the averaged exchange field seen by the STI surface states in the presence of ferromagnetically aligned impurity spins which point perpendicular to the surface. The dashed curve is the LDOS in the absence of the local impurity perturbation, which exhibits an energy gap between $\pm\Delta$. We see that when the ratio $U(J_{\text{eff}}\mathbf{S})^{-1}$ is greater than a certain value (equal to 1 for no energy broadening), bound states appear inside the energy gap. Thus, experimental measurements of the LDOS will may not observe a clear spectral gap, even though the mobility gap is nonzero, because the inter-gap bound states do not contribute to transport.

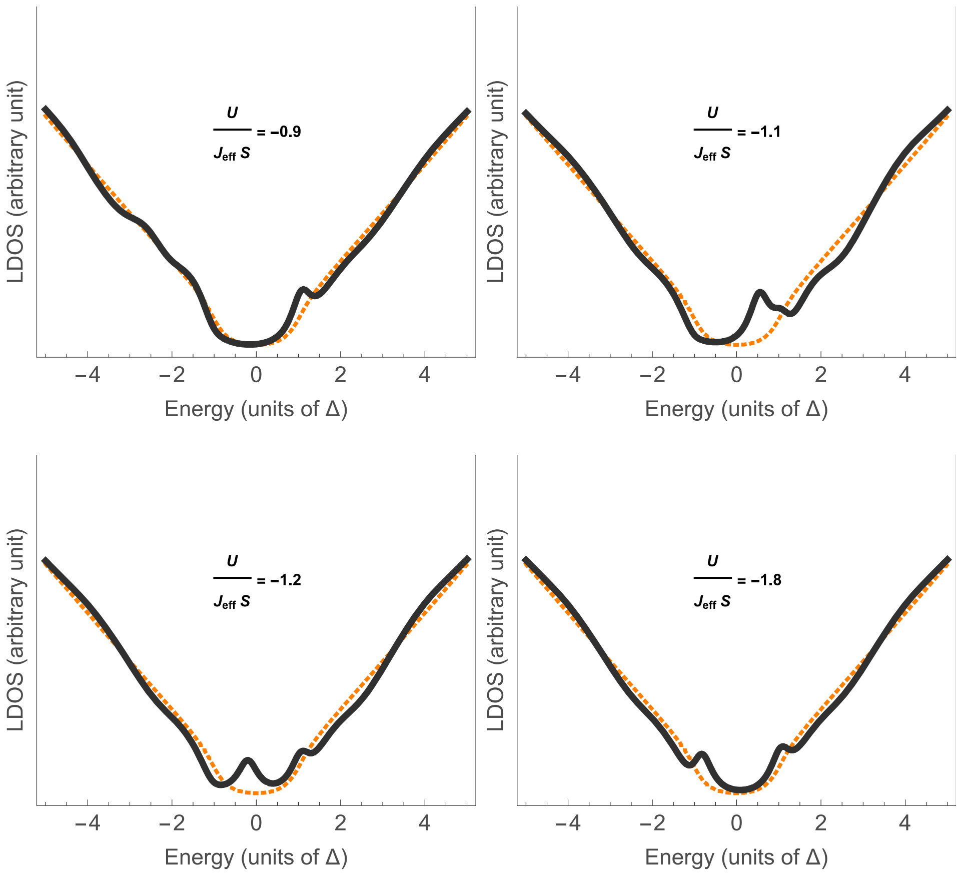

Supplementary Figure 2: The same as Fig S1, but with negative values of the ration $U(J_{\mathrm{eff}}\mathbf{S})^{-1}$. The spectra in Supplementary Figures 1 and 2 are related by reflection about the Dirac point energy.

Supplementary Figure 3 – Raw STS data before zero-field background subtraction

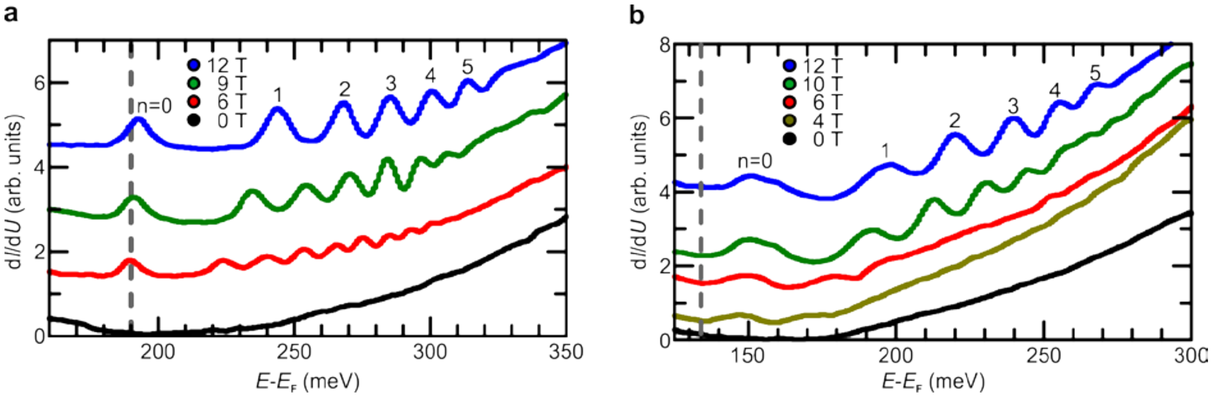

Supplementary Figure 3: Scanning tunneling spectroscopy data obtained on (**a**) pristine and (**b**) V-doped Sb_2Te_3 at different magnetic fields applied perpendicular to the sample surface. Spectra at different magnetic fields have been vertically shifted for clarity. For sufficiently large fields, a well-defined sequence of peaks emerges form the background, signaling the condensation of the two-dimensional topological electron gas into Landau orbits. The index n identifies the Landau level order. The dashed gray line marks the position of the Dirac point as expected for linearly dispersing massless particles. In V-doped samples, its shift with respect to the position of the first peak ($n = 0$) unequivocally signals the emergence of a mass term in the Dirac spectrum.

Supplementary Notes:

Supplementary Note 1 - Theoretical modeling

We start with a general low energy effective Hamiltonian of strong topological insulator (STI) surface electronic states, in the presence of impurities that have both local magnetic and potential scattering components [1,2],

$$\mathcal{H} = \left(\mathbf{k}_x\boldsymbol{\sigma}_x + \mathbf{k}_y\boldsymbol{\sigma}_y\right) + \sum_{\text{imp}}[J\mathbf{S}_i \cdot \boldsymbol{\sigma}(\mathbf{r}_i) + Un(\mathbf{r}_i)]. \quad (1)$$

Here, J and U are the impurity magnetic exchange and potential strengths, respectively, while $\mathbf{k}$ is the wave vector. $n(\mathbf{r}_i)$ and $\boldsymbol{\sigma}(\mathbf{r}_i)$ are the electronic number and spin densities, respectively, at the location, $\mathbf{r}_i$, of the ith impurity. We have chosen units such that the Planck's constant, $\hbar$, and the Fermi (Dirac) velocity, v_{D}, are both unity.

When the impurities align ferromagnetically perpendicular to the surface (z-direction), due to RKKY interactions [1] or other effects, it is usually assumed that an energy gap appears at the Dirac point due to the spatially averaged carrier-impurity exchange interaction. However, this analysis misses the role of impurity potential scattering, that lead to intragap resonances [1,2]. Taking the potential part into account, the effective Hamiltonian, including a mean field gap arising from the magnetic moments, becomes

$$\mathcal{H}_{\text{eff}} = \left(\mathbf{k}_x\boldsymbol{\sigma}_x + \mathbf{k}_y\boldsymbol{\sigma}_y + \Delta\boldsymbol{\sigma}_z\right) + \sum_{\text{imp}}[J_{\text{eff}}\mathbf{S} \cdot \boldsymbol{\sigma}_z + U]\, n(\mathbf{r}_i). \quad (2)$$

The mean field Dirac mass,

$$\Delta \simeq J\sum_{\text{imp}}\langle n(\mathbf{r}_i)(\mathbf{S}_i)_z\rangle, \quad (3)$$

is half the energy gap of the (now massive) surface Dirac states, calculated using an appropriate averaging procedure over the impurity-carrier exchange interactions. J_{eff} is the residual effective magnetic impurity strength of the impurity acting on these *massive* Dirac fermions.

Ignoring the chemical potential shift, a mean field effect arising from potential scattering, as well as residual local impurity effects, these massive Dirac fermions possess a fully gapped spectrum:

$$E(k) = \pm\sqrt{\Delta^2 + k^2}. \quad (4)$$

However, as we show now, completely ignoring the local residual effects of potential scattering is a mistake. In general, no magnetic impurity should have $U = 0$. The inclusion of U leads to the formation of impurity resonances in gapless Dirac Materials [1,3]. Numerical analysis [2] shows that in the presence of both magnetic and potential scattering, the energy spectrum contains both a gapped feature due to magnetism, as well as the aforementioned impurity resonances due to potential scattering. As a result, the spectrum is not fully gapped, a qualitative difference from the mean field result.

Using the effective Hamiltonian, Supplementary Eq. (2), in the presence of a single impurity, we can exactly calculate the single particle Green's function using the T-matrix technique [1,4]. We find analytically that the T-matrix is singular, i.e., there exists a bound state, when the energy, E, is a solution to the equation,

$$g(E)(J_{\text{eff}}\mathbf{S} \pm U)(\Delta \pm E) = 1, \quad (5)$$

while also being inside the gap, $-\Delta < E < \Delta$. $g(E)$ is the on-site Green's function, which for $-\Delta < E < \Delta$ is the following

$$g(E) = -\frac{1}{4\pi}\ln\left(1+\frac{\Lambda^2}{\Delta^2-E^2}\right) < 0. \quad (6)$$

Λ is the high energy cutoff. These equations always possess a solution when U is larger than $J_{\text{eff}}\mathbf{S}$ in magnitude, and so there is a inter-gap bound state for a range of impurity parameters.

Including appropriate spectral broadening of the Dirac states, as is observed experimentally, we present the calculated spectra for a range of values of $U(J_{\text{eff}}\mathbf{S})^{-1}$, in Supplementary Figures 1 and 2. For these calculations we have used units in which the Planck's constant, $\hbar$, the Fermi velocity, v_{D}, as well as the mean field energy gap, Δ, are unity. We have used the values $\Lambda = 50$, $J_{\text{eff}}\mathbf{S} = 10$ and the spectral broadening, $\Gamma = 0.3$. Impurities are assumed to be located at distances $r = 0.6$, 1.4, 1.5, 1.6, 1.8, 1 and 3; these locations are chosen randomly to yield LDOS that resemble Fig. 2d in the main text and are consistent with the neighborhood of the location where that data was measured. Multiple impurity scattering has been ignored. As U becomes large in magnitude compared to $J_{\text{eff}}\mathbf{S}$, a bound state peak appears inside the energy gap.

Our calculations thus directly prove that for a range of reasonable parameters, (when the magnitude of U is larger than that of $J_{\text{eff}}\mathbf{S}$, for $\Gamma = 0$), while the spectrum of *extended* states may possess an energy gap (the mobility gap), the LDOS need not exhibit an energy gap. This reflects the competing trends between gap opening due to magnetic coupling, and gap filling due to localized states seeded from the scalar potential-induced spectral resonances in the gapless case.

Supplementary References:

[1] Biswas, R. R. & Balatsky, A. V. Impurity-induced states on the surface of three-dimensional topological insulators. *Phys. Rev. B* **81**, 233405 (2010).

[2] Black-Schaffer, A. M., Balatsky, A. V. & Fransson, J. Filling of magnetic-impurity-induced gap in topological insulators by potential scattering. *Phys. Rev. B* **91**, 201411(R) (2015).

[3] Wehling, T. O., Black-Schaffer, A. M. & Balatsky, A. V. Dirac materials Adv. Phys. 63, 1-76 (2014).

[4] Doniach, S. & Sondheimer, E. H. *Green's functions for solid state physicists* (Imperial College Press, 1998).
